# Hyperintense acute reperfusion marker (HARM) and thrombus analysis in acute ischemic stroke

**DOI:** 10.3389/fneur.2026.1723099

**Published:** 2026-03-19

**Authors:** Sena Aksoy, İbrahim Kulaç, Hatem Hakan Selçuk, Batuhan Kara, Ali Burak Kızılırmak, Bayram Yılmaz, Yasemin Gürsoy Özdemir, Atay Vural, Aysun Soysal

**Affiliations:** 1 Department of Neurology, Bakırköy Prof. Dr. Mazhar Osman Mental Health and Neurological Diseases Training and Research Hospital, University of Health Sciences, Istanbul, Türkiye; 2 Department of Pathology, Koc University, Istanbul, Türkiye; 3 Department of Neuroradiology, Bakirkoy Dr. Sadi Konuk Training and Research Hospital, Istanbul, Türkiye; 4 Research Center for Translational Medicine, Koc University, Istanbul, Türkiye; 5 Department of Physiology, Yeditepe University, Istanbul, Türkiye; 6 Department of Physiology, Dokuz Eylül University, İzmir, Turkey; 7 Department of Neurology, Koc University, Istanbul, Türkiye

**Keywords:** acute ischemic stroke, blood–brain barrier, hyperintense acute reperfusion marker, mechanical thrombectomy, thrombus analysis

## Abstract

Mechanical thrombectomy allows thrombus analysis in acute ischemic stroke. The extravasation of contrast material into the cerebrospinal fluid (CSF) on fluid-attenuated inversion recovery (FLAIR) imaging is referred to as a hyperintense acute reperfusion marker (HARM), which indicates deterioration of the blood–brain barrier (BBB). In this study, we aimed to examine the relationship between the histopathological features of thrombi and HARM in acute ischemic stroke. A total of 56 patients who underwent mechanical thrombectomy (MT) were included in the study. Procedural data, including the number of passes, techniques used, and recanalization scores based on the modified Thrombolysis in Cerebral Infarction (mTICI) scale, were documented. FLAIR imaging was performed 24 h after contrast administration. The presence of contrast extravasation into the cerebral sulci was defined as a positive HARM. A total of 52 thrombi were successfully retrieved and analyzed. Thrombus sections were stained with hematoxylin and eosin (H&E) to evaluate their fibrin and erythrocyte composition. Immunohistochemical staining using CD3, CD20, and CD45 antibodies was performed to identify T lymphocytes, B lymphocytes, and total leukocytes, respectively. In our study, it was found that clinical outcomes—measured by National Institutes of Health Stroke Scale (NIHSS) scores at 24 h and modified Rankin Scale (mRS) scores at 90 days—were significantly worse in HARM-positive patients. A histopathological analysis revealed that thrombi from HARM-positive patients were predominantly rich in fibrin, suggesting a potential association between thrombus composition and BBB disruption. Future studies incorporating more detailed thrombus characterization alongside advanced radiological markers may yield valuable insights into stroke pathophysiology.

## 1 Introduction

Stroke is a leading cause of mortality and morbidity worldwide (1, 2). During cerebral ischemia, the affected brain tissue can be broadly divided into two zones: the ischemic core, which is irreversibly damaged due to severely reduced blood flow, and the surrounding penumbra, in which perfusion is reduced but still sufficient to preserve cellular viability for a limited period. The penumbra represents salvageable tissue and is the primary target of acute stroke interventions. Large multicenter clinical trials have demonstrated the efficacy of both intravenous thrombolysis (IV tPA) and mechanical thrombectomy in restoring perfusion and improving outcomes by rescuing penumbral tissue (1, 3).

Despite successful recanalization, some patients may not experience favorable clinical outcomes. This can occur when adequate tissue reperfusion is not achieved despite the restoration of vessel patency. The mismatch between recanalization and poor clinical outcome can be attributed to factors such as microvascular injury, impaired collateral circulation, or blood–brain barrier disruption, which prevent effective reperfusion at the tissue level and may lead to hemorrhage (4, 5). Although several studies have investigated the mechanisms of ischemia and reperfusion and potential biomarkers for stroke, the exact underlying mechanism remains to be understood (6, 7).

The blood–brain barrier is disrupted in conditions such as infection, malignancy, and stroke, which can be detected by the passage of contrast material into brain tissue on magnetic resonance imaging (MRI). In acute ischemic stroke, BBB disruption can be identified by the presence of contrast material in CSF on early contrast-enhanced FLAIR imaging, referred to as a hyperintense acute reperfusion marker (8, 9). HARM positivity is associated with intracerebral hemorrhage and poor prognosis following mechanical thrombectomy (8).

Mechanical thrombectomy provides an oppurtunity for the examination of the retrieved thrombi. Several studies exist on the histopathological and immunohistochemical examinations of thrombi and their possible correlations with stroke type or treatment outcomes (10–20). A better understanding of thrombus characteristics and underlying pathophysiology may offer valuable insights for optimizing recanalization strategies and improving clinical outcomes in patients with acute ischemic stroke.

In this study, we aimed to examine HARM, histopathological features of thrombus, and their potential relationships with stroke etiology and clinical outcome. Although there are studies in the literature on the relationship between HARM and clinical outcomes following mechanical thrombectomy, to our knowledge, this is the first study examining HARM and thrombus analysis.

## 2 Materials and methods

A total of 56 patients who were admitted to the hospital with acute ischemic stroke and underwent mechanical thrombectomy were included in the study. Patients were recruited after written consent was obtained. The primary outcome variables were the 90-day mRS score and HARM positivity. The secondary outcome variable was intracranial hemorrhage. The exclusion criteria included contraindications for MRI with contrast enhancement, such as the presence of MRI-incompatible implanted devices, kidney failure, or contrast allergy.

Ethical approval for this study was obtained from Bakırköy Dr. Sadi Konuk Training and Research Hospital Clinical Research Ethics Committee on 07.12.2020 with decision number 2020–24-16.

### 2.1 Clinical information

Demographic characteristics and detailed clinical data were collected for all patients, including baseline NIHSS scores and time intervals such as symptom-to-door, symptom-to-puncture, and symptom-to-recanalization times. Laboratory parameters such as leukocyte and neutrophil counts, along C-reactive protein (CRP) levels, were also recorded (21) (Table 1). Procedural data were collected, including duration of the thrombectomy, number of passes, technique used, and recanalization score using the mTICI (22). Clinical outcomes were evaluated using NIHSS scores at 24 h and at discharge, and mRS scores at discharge and at 90 days post-procedure (21, 22). For the assessment of mRS, patients were categorized into three groups based on functional independence and mortality: mRS 0–2, mRS 3–5, and mRS 6. Stroke etiology was determined based on the Trial of Org 10,172 in Acute Stroke Treatment (TOAST) classification following a comprehensive etiological workup (23).

**Table 1:** Baseline characteristics, procedural details, and clinical outcomes of patients.

	MT (*n* = 39)	iv tPA+MT (*n* = 17)	All (*n* = 56)
*Baseline characteristics*			
Age (mean, SD)	66 (13.37)	62.8 (19.13)	65 (15.23)
Female (n, %)	20 (51.3%)	9 (52.9%)	29 (51.8%)
Diabetes (n, %)	11 (28.2%)	8 (47.1%)	19 (33.9%)
Hypertension (n, %)	19 (48.7%)	9 (52.9%)	28 (50%)
Hyperlipidemia (n, %)	16 (41%)	7 (41.2%)	23 (41.1%)
Previous stroke (n, %)	8 (20.5%)	3 (17.6%)	11 (19.6%)
Atrial fibrillation (n, %)	14 (35.9%)	5 (29.4%)	19 (33.9%)
Onset NIHSS	12 (10–15)	10 (8–13)	11 (9–14)
*Timemetric parameters*			
Symptom-to-door (min)	61.5 (40.75–260.75)	100 (60–142)	68 (43–220)
Symptom-to-puncture (min)	265.5 (170.5–431.25)	234 (160–258)	235 (169–354)
Symptom-to-recanalization (min)	295.5 (191.25–462.25)	257 (188–280)	260 (190–388)
Procedure time (min)	34 (19–58)	26 (17.5–36.5)	28 (19.25–52.5)
*Procedural data*			
Number of passes	1 (1–3)	1 (1–2.5)	1 (1–2.75)
Stent-retriever (n, %)	22 (61.1%)	10 (58.8%)	32 (60.4%)
Aspiration (n, %)	10 (27.8%)	4 (23.5%)	14 (26.4%)
Combined (n, %)	4 (11.1%)	3 (17.6%)	7 (13.2%)
*Clinical outcome*			
NIHSS at 24 h	9 (4-14)	6 (3.5-10)	8 (4-12)
NIHSS at discharge	5 (2-10)	4 (0.5-8)	5 (1.5-10)
mRS at 90 days			
mRS 0-2 (n, %)	20 (51.3%)	12 (70.6%)	32 (57.1%)
mRS 3-5 (n, %)	11 (28.2%)	3 (17.6%)	14 (25%)
mRS 6 (n, %)	8 (20.5%)	2 (11.8%)	10 (17.9%)

### 2.2 Neuroimaging

All patients underwent imaging using a CT scanner (Somatom Definition Flash, Siemens). MRI was performed using 1.5-T scanners (Ingenia, Philips Healthcare, Best, the Netherlands, or Magnetom Aera, Siemens, Munich, Germany).

The presence of the hyperdense artery sign (HAS) was assessed on baseline CT images. In patients with hyperdense artery sign, clot attenuation was measured in Hounsfield units (HU). Since differences in hematocrit levels may affect blood density, thrombus density was evaluated in relation to the contralateral middle cerebral artery using the formula “HU clot/ HU contralateral middle cerebral artery” (24).

Carotid MR angiography was performed at 24 h as part of the etiological examination. During MR angiography, 15 mL of gadolinium (Dotarem, Guerbet) was administered intravenously. Fluid-attenuated inversion recovery (FLAIR) imaging was performed 24 h after gadolinium administration, according to the protocol of field of view (FOV) 240 mm × 218 mm, matrix 252 mm × 153 mm, section thickness 5 mm, repetition time (TR) 11,000 ms, and echo time (TE) 140 ms. Contrast extravasation in the sulcus on FLAIR imaging was considered positive for HARM. Radiological evaluation for HARM was performed by two radiologists blinded to clinical data.

Infarct volume and intracranial hemorrhage volume were calculated using the ABC/2 method based on MRI or CT images obtained at 24-h post-treatment (25).

### 2.3 Thrombus analysis

After gently rinsing with saline, the retrieved thrombus samples were collected and fixed in a 10% formaldehyde solution for at least 24 h and up to a maximum duration of 7 days (26). After fixation, thrombi were processed for paraffin embedding, sectioning, and hematoxylin–eosin (H&E) staining for the examination of fibrin, red blood cells (RBCs), and white blood cells (WBCs) (Supplementary material). For the analysis of thrombi, one section from each sample, representing the whole thrombus, was stained using each staining method (27). Quantification of RBCs and fibrin was performed by a histopathological analysis. Immunohistochemical staining for CD3, CD20, and CD45 was performed to identify and quantify T lymphocytes, B lymphocytes, and total leukocyte populations, respectively (Supplementary material).

**Figure 1:**
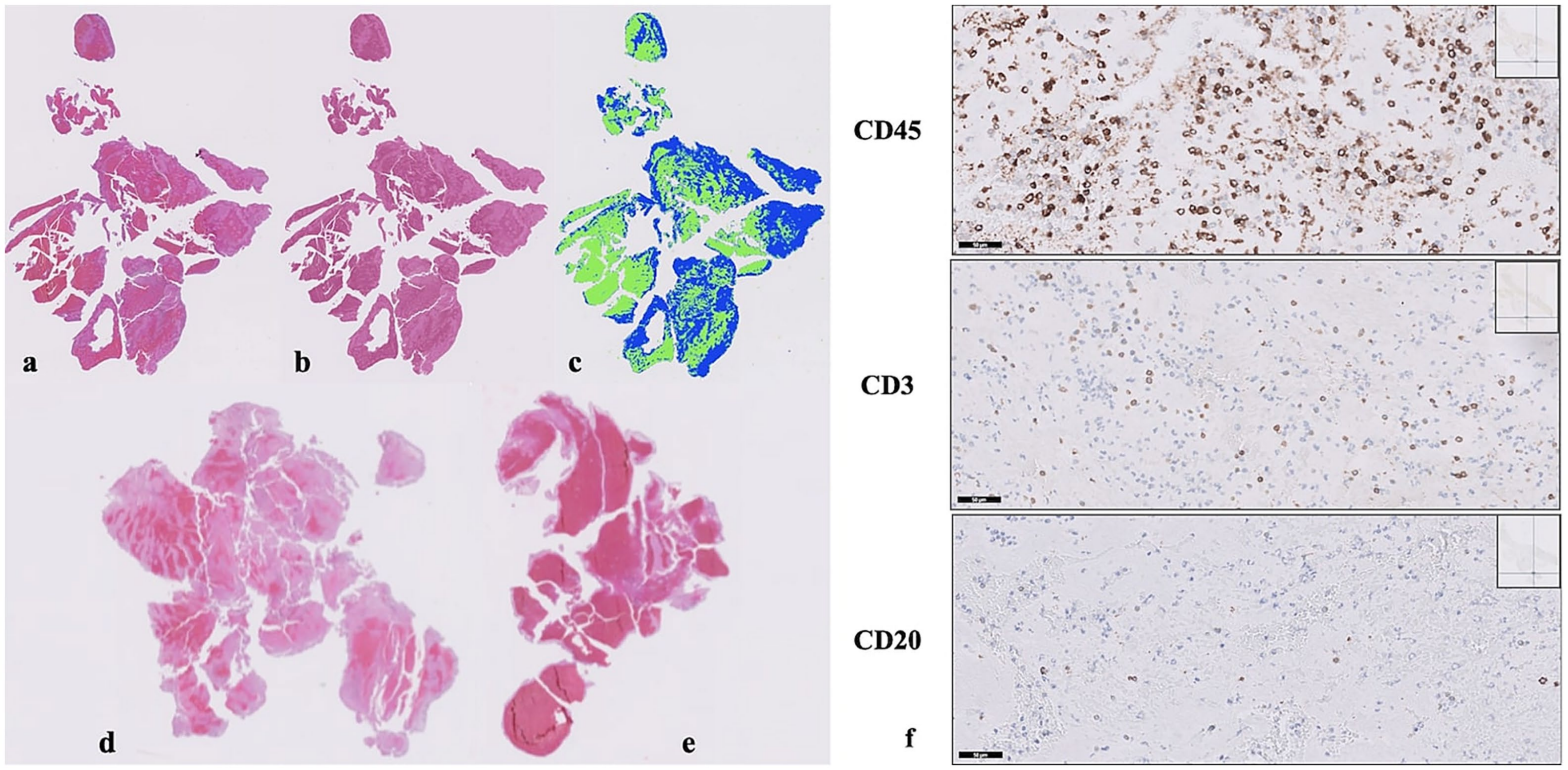
Representative histopathological and immunohistochemical findings of retrieved thrombi. H&E staining: pink-red areas show RBCs; light pink areas show fibrin **(a)**; thrombus after color-based segmentation with Fiji: pink-red areas show RBCs; light pink areas show fibrin **(b)**; thrombus after color-based segmentation with Orbit: green areas show RBCs; blue areas show fibrin **(c)**; examples of fibrin-rich **(d)** and RBC-rich **(e)** thrombi. Immunohistochemical staining, thrombus stained with CD45, CD3, and CD20 (×20 magnification) **(f)**.

Orbit (Version 3.64) and Fiji (Version 1.0) were used for image analysis. The analysis of thrombi stained with H&E was performed using a color-based segmentation method to determine the relative quantitative fraction of RBCs, WBCs, and fibrin based on the area they occupied within the thrombus in both Orbit and Fiji (Figures 1a–c). In the quantitative analysis of immunohistochemical staining with CD3, CD20, and CD45, five areas with the highest cell density at x20 magnification were selected for each sample, and cell counting was performed manually using the CellCounter plugin of Fiji (Figure 1f).

### 2.4 Statistics

Statistical analyses were carried out using appropriate parametric and non-parametric tests according to the distribution of the data. Non-parametric data were expressed as median value and interquartile range, and parametric data were expressed as mean and standard deviation. Differences between groups were analyzed by using Student’s *t*-test or the Mann–Whitney U-test, and correlation analyses were performed with Pearson’s or Spearman’s tests. Fisher’s exact test was utilized to evaluate categorical data. A *p* < 0.05 was considered statistically significant. SPSS (Version 20) and GraphPad (Version 9.1.2) programs were used as statistical analysis programs.

## 3 Results

A total of 56 patients were included in this study. Mechanical thrombectomy was performed in patients with proximal large vessel occlusion who presented within 6 h of symptom onset or patients with clinical diffusion mismatch. Mechanical thrombectomy was performed in 39 (69.6%) of 56 patients, and both IV tPA and mechanical thrombectomy were performed in 17 (30.4%) patients (Table 1).

Successful recanalization (TICI 2b-3) was achieved in 44 (78.6%) patients. Patients with an mRS score of 0–2 were evaluated as functionally independent. A total of 32 (57.2%) patients were functionally independent at 90 days, and 10 (17.9%) patients died.

**Figure 2:**
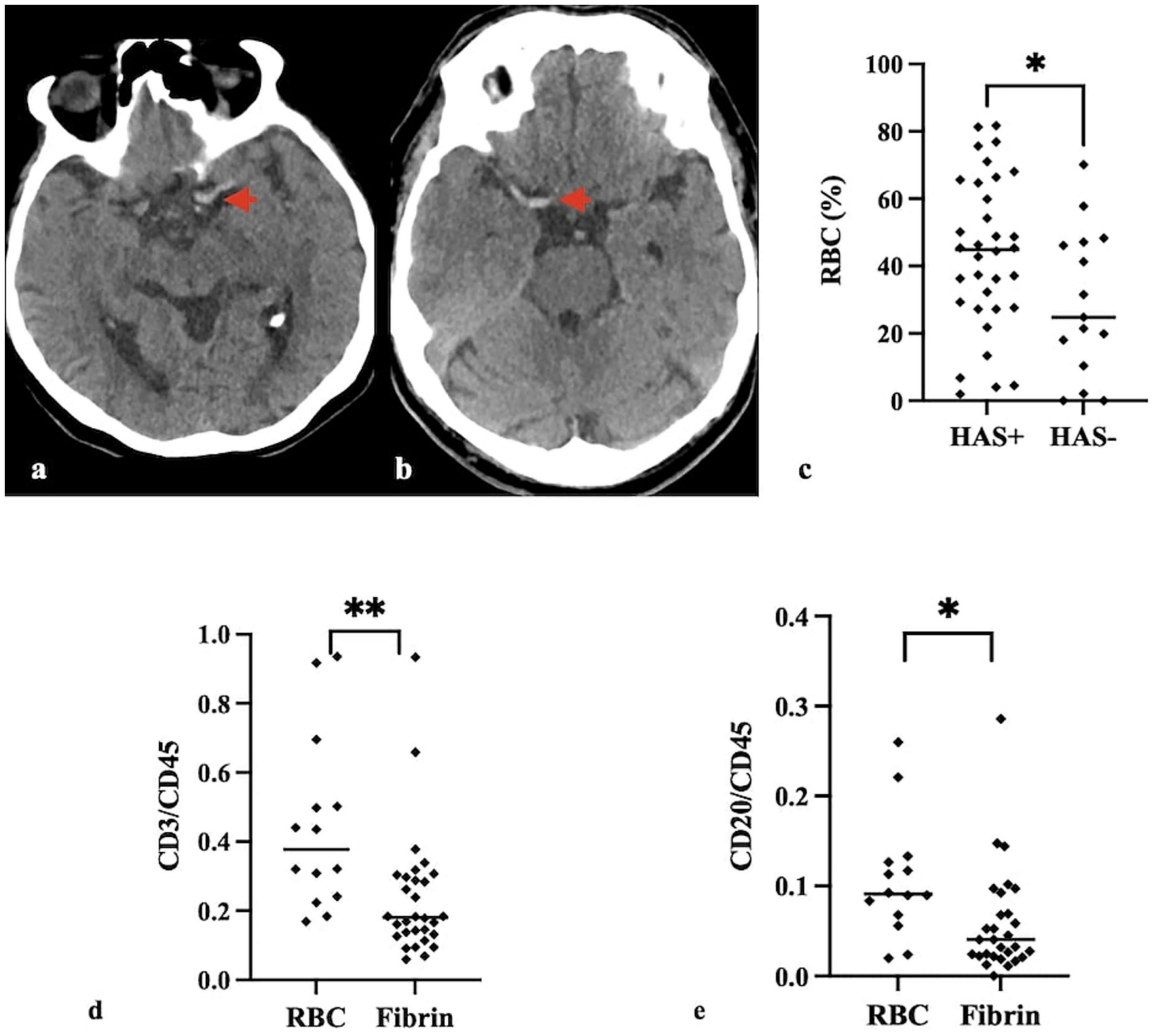
Hyperdense artery signs on non-contrast cranial CT on left MCA **(a)** and right MCA **(b)**. Quantitative analyses show that the RBC ratio in thrombi of HAS-positive patients was higher than in thrombi of HAS-negative patients (*p* = 0.046) **(c)**. The CD3/CD45 **(d)** and CD20/CD45 **(e)** ratios were higher in RBC-rich thrombi compared to fibrin-rich thrombi (*p* = 0.001, *p* = 0.011, respectively). *p < 0.05 **p < 0.01.

Hyperdense artery signs were observed in 36 (65.5%) patients (Figures 2a,b). Among the 11 patients who developed hemorrhage, 1 was classified as hemorrhagic infarction, 5 as parenchymal hematoma type 1, 3 as parenchymal hematoma type 2, and 2 as subarachnoid hemorrhage.

According to the TOAST classification, 18 (32.1%) patients had large artery atherosclerosis, 24 (42.9%) patients had cardioembolism, 1 (1.8%) had stroke due to other etiologies, and 13 (23.2%) had cryptogenic stroke.

A total of 52 thrombi were stained with H&E and analyzed using the color-based segmentation method with the Fiji and Orbit software programs. Thrombi were categorized into two groups based on their composition: those with fibrin comprising more than 50% of the thrombus area were classified as fibrin-rich, while thrombi with red blood cells exceeding 50% of the area were classified as RBC-rich (Figures 1d,e). A total of 37 (71.2%) thrombi were considered fibrin-rich, and 15 (28.8%) were RBC-rich. No significant difference was observed in the rates of fibrin-rich or RBC-rich thrombi between the Fiji and Orbit image analysis programs. Furthermore, a strong positive correlation was found between the fibrin and RBC ratios calculated by the two software platforms (*p* < 0.001).

No statistically significant association was observed between stroke etiology and the dominant thrombus component. Additionally, no significant relationship was found between clinical outcome parameters and RBC/fibrin dominance.

A significant correlation was observed between the RBC ratio and the hyperdense artery sign, with higher RBC ratios in HAS-positive patients (*p* = 0.046) (Figure 2c).

CD3, CD20, and CD45 stainings were performed on 49 thrombi (Figure 1f), and the absolute counts as well as the CD3/CD20, CD3/CD45, and CD20/CD45 ratios were determined. No relationship was observed between CD3/CD20, CD3/CD45, and CD20/CD45 ratios and time metric parameters, lesion and hemorrhage volumes, and stroke etiology.

CD3/CD45 and CD20/CD45 ratios were negatively correlated with the fibrin ratio (*p* = 0.006, *p* = 0.004) and positively correlated with the RBC ratio (*p* = 0.006, *p* = 0.004). Similarly, CD3/CD45 and CD20/CD45 ratios were higher in RBC-rich thrombi (*p* = 0.001, *p* = 0.011) (Figures 2d,e).

**Figure 3:**
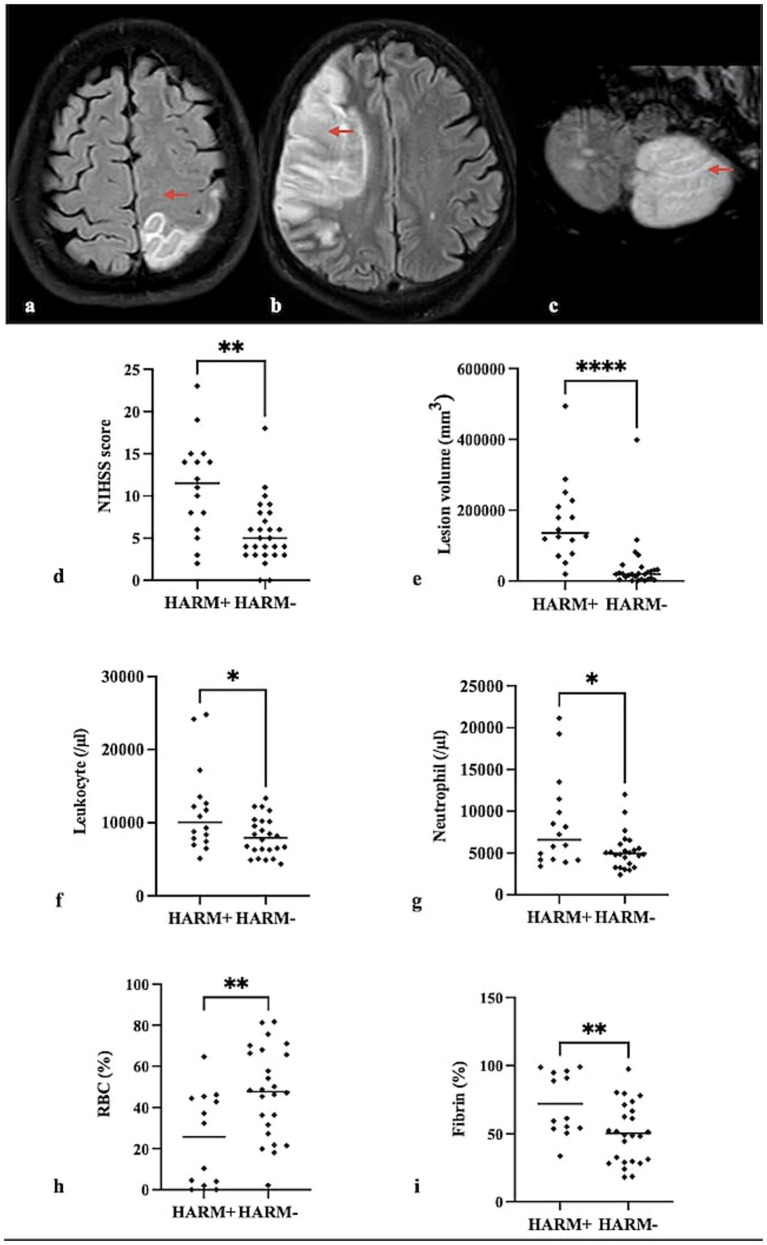
Representative MRI images demonstrate HARM (a–c, arrows). In axial FLAIR imaging with contrast, there is a hyperintense area in the MCA-ACA supplying area in the left posterior frontal and parietal lobes, and hyperintensity is compatible with contrast agent extravasation between the sulci **(a)**, a large hyperintense infarct area in the MCA supplying area in the right frontal lobe, and hyperintensity of contrast agent extravasation between the sulci **(b)**, infarction in the posterior inferior cerebellar artery supplying area in the left cerebellum, and the contrast extravasation between the cerebellar folios **(c)**. Quantitative analyses showed that NIHSS score **(d)** and lesion volume **(e)** at 24 h in HARM-positive patients were higher than in HARM-negative patients (*p* = 0.002, *p* < 0.0001). The blood leukocyte **(f)** and neutrophil **(g)** counts were higher in HARM-positive patients (*p* = 0.025, *p* = 0.047). In the thrombi of HARM-positive patients, a lower ratio of RBCs **(h)** and a higher ratio of fibrin **(i)** were found (*p* = 0.005, *p* = 0.007). *p < 0.05 **p < 0.01 ****p < 0.0001.

Contrast extravasation in the sulcus on FLAIR imaging 24 h after contrast agent injection was considered positive for HARM (Figures 3a–c). In 13 of 56 patients, HARM could not be evaluated due to a medical condition that prevented MRI, such as intubation. HARM was not observed in 27 (62.8%) of the remaining 43 patients, while HARM was positive in 16 (37.2%) patients. The mean baseline NIHSS score was 11.6 ± 3.3 in patients in whom HARM assessment could be performed compared with 13 ± 5.1 in those in whom it could not be performed (*p* = 0.43). When clinical outcomes were evaluated, the mean 24-h NIHSS score was 7.6 ± 5.2 in patients with evaluable HARM, whereas it was 12 ± 7.2 in patients without evaluable HARM (*p* = 0.17). At 90 days, 65.1% of patients with evaluable HARM were functionally independent compared with 30.8% of patients in whom HARM assessment could not be performed.

**Figure 4:**
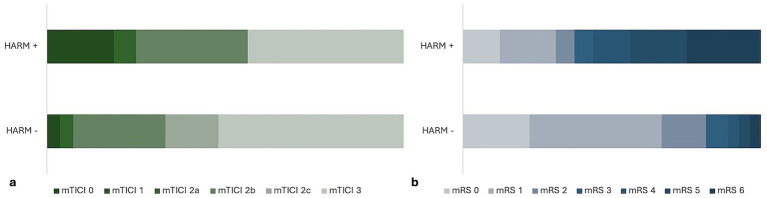
Comparison of angiographic and functional outcomes in HARM-positive and HARM-negative patients. HARM-positive patients demonstrated lower rates of favorable reperfusion (mTICI 2b–3) **(a)** and a lower proportion of good functional outcomes (mRS 0–2) **(b)** compared with HARM-negative patients.

There was no significant relationship between baseline NIHSS scores and HARM positivity, but NIHSS scores at 24 h were higher in HARM-positive patients (*p* = 0.22, *p* = 0.002) (Figure 3d; Table 2). No significant correlation was found between HARM and time metric parameters, procedure time, number of passes, or mechanical thrombectomy method (Table 2). HARM-positive patients demonstrated lower rates of favorable reperfusion (mTICI 2b-3), but it was not statistically significant (*p* = 0.12) (Figures 4a).

**Table 2:** Comparison of clinical, procedural, and laboratory variables between HARM-positive and HARM-negative patients.

	HARM+ (*n* = 16)	HARM- (*n* = 27)	*p*-value
Symptom-to-door (min)	62 (39.75–247)	122.5 (42.25–212.5)	0.619
Symptom-to-puncture (min)	195 (166.75–380.75)	258 (157.5–335.25)	0.936
Symptom-to-recanalization (min)	216.5 (186.25–408.75)	291 (180.75–367.5)	0.689
Procedure time (min)	27.5 (16.25–60.75)	24 (19–36)	0.481
Number of passes	1 (1–2)	1 (1–2)	0.366
NIHSS at admission	12.5 (9.25–15.75)	11 (9–13)	0.22
NIHSS at 24 h	11.5 (6.5–14.75)	5 (3–8)	0.002^**^
Leukocyte number (/μL)	10,080 (7542–13,332)	7,950 (6302–10,222)	0.025^*^
Neutrophil number (/μL)	6,590 (4207–11,067)	4,960 (3400–5,932)	0.047^*^
Lesion volume (cm^3^)	135.9 (87–222.9)	19.49 (5.46–31.52)	<0.0001^****^

*p<0.05, **p<0.01, ****p<0.0001.

Lesion volume at 24 h was significantly higher in HARM-positive patients (*p* < 0.0001) (Figure 3e; Table 2). No significant relationship was observed between HARM and the presence of hemorrhage or hemorrhage volumes. A higher rate of HARM positivity was observed in patients with higher mRS scores at 90 days (*p* = 0.002) (Figure 4b). It was also found that blood leukocyte and neutrophil counts were higher in HARM-positive patients (*p* = 0.025, *p* = 0.047) (Figures 3f,g; Table 2).

A significant correlation was observed between the fibrin and RBC ratios in thrombi and HARM. In HARM-positive patients, the fibrin ratio was higher and the RBC ratio was lower (*p* = 0.007, *p* = 0.005) (Figures 3h,i). No significant correlations were observed between HARM and CD3/CD20 or between CD3/CD45 and CD20/CD45 ratios.

## 4 Discussion

Studies on thrombus histopathology have recently increased with the widespread use of mechanical thrombectomy. Various staining techniques and image analysis methods have been applied to explore potential associations between thrombus composition and clinical or imaging findings (27, 28). For thrombus analysis, fixation for 24 h followed by the embedding process is generally recommended. Prolonged fixation exceeding 1 week may adversely affect subsequent staining by irreversibly masking target antigens and increasing the autofluorescence of clot material (27). In the present study, fixation was typically performed for 24–48 h, and no specimens were fixed for longer than 7 days; therefore, the fixation duration is unlikely to have significantly influenced the staining quality.

H&E-stained images were analyzed using two different programs, Fiji and Orbit, to explore potential methodological differences. No differences were observed in fibrin-rich or RBC-rich thrombus proportions between the two programs. In addition, a strong correlation was found between the fibrin and RBC ratios calculated with Fiji and Orbit. However, the Orbit program enables more thrombus analysis in a shorter time by providing ease of use, as it allows better visualization of different structures with different colors and the creation of models with machine learning while performing the analysis.

Recent studies on thrombus histopathology generally focus on the relationship between thrombus structure and stroke etiology. In the literature, there are studies showing that the rates of fibrin-platelet-rich thrombus are higher in cardioembolic stroke (12, 16). Similarly, the higher rate of fibrin-platelet dominance in cryptogenic stroke suggested that cryptogenic stroke may be of cardioembolic origin (12, 29). However, other studies have failed to demonstrate a clear association between thrombus composition and stroke etiology (14). In a recent review, it was stated that a clear relationship between stroke etiology and thrombus structure could not be demonstrated (30). These discrepancies may be attributable to differences in staining techniques, image analysis methodologies, and limited sample sizes across studies. In this context, the present findings should be interpreted as exploratory and hypothesis-generating rather than definitive.

The hyperdense artery sign is important for the visualization of thrombus on non-contrast CT. Studies have shown that the hyperdense artery sign is associated with a high erythrocyte ratio in the thrombus (31, 32). In our study, consistent with previous studies, it was found that the ratio of erythrocytes in the thrombus was higher in HAS-positive patients. However, no association was observed between HAS and the thrombectomy technique used, a finding that should be interpreted cautiously given the limited sample size.

In the present study, CD3/CD45 and CD20/CD45 ratios were found to be positively correlated with red blood cell and fibrin content. Although these associations do not establish causality, they suggest a potential association between thrombus structural dominance and leukocyte subtype composition. In line with previous studies demonstrating preferential leukocyte localization within platelet–fibrin–rich regions of thrombi, these preliminary findings underscore the need for more comprehensive studies to further elucidate the interplay between thrombus composition and neuroinflammatory mechanisms in the pathophysiology of stroke (33).

Exploratory analyses of clinical outcomes have demonstrated that HARM-positive patients had higher NIHSS scores and lesion volumes at 24 h, along with higher 90-day mRS scores. The lack of an association with baseline NIHSS scores may indicate that the observed differences in clinical outcomes could be related, at least in part, to neuroinflammatory processes developing within the first 24 h, as well as to potential deterioration of the BBB. There are studies supporting the relationship of HARM with early blood–brain barrier deterioration and poor prognosis for acute ischemic stroke (8, 34–36). However, unlike some prior reports, no relationship was found between HARM and hemorrhagic transformation or hemorrhage volumes in our study. Methodological differences across studies may partly explain these discrepancies. Although our study included only patients who underwent mechanical thrombectomy, other studies had a wider window for patient selection, enrolling patients who presented within 24 h of symptom onset or with transient ischemic stroke (34–36). There are also differences in the time from contrast injection to FLAIR imaging between studies. Contrast material-enhanced FLAIR imaging has been reported to be performed within 2–48 h after contrast administration. Longer time intervals may allow contrast material leaking through blood–brain barrier disruption to gradually accumulate in the subarachnoid space and reach a sufficient concentration, potentially leading to a higher rate of HARM detection (36). Although no statistically significant difference in hemorrhage rates was observed in HARM-positive patients in our study, the observed increases in 24-h NIHSS scores and lesion volume may be interpreted as suggesting a possible association between BBB disruption and ischemic injury, potentially through mechanisms other than hemorrhage.

The leukocyte and neutrophil counts were higher in HARM-positive patients. In recent studies, high neutrophil counts were associated with poor prognosis in patients with successful recanalization with endovascular treatment. The mechanism that causes this situation might be impaired microcirculation and thromboinflammation (37, 38). This finding may cautiously be interpreted as suggesting a possible association between early blood–brain barrier deterioration identified by HARM and neuroinflammatory processes; however, this relationship warrants further investigation.

The limitations of the study are the small number of patients and the inability to include some patient groups that are not suitable for MRI. In addition, some patients could not undergo a second MRI scan due to clinical deterioration during follow-up, such as the need for intubation, which may have resulted in the underrepresentation of patients with more severe strokes and introduced potential selection bias.

In conclusion, this study suggests that the fibrin and RBC content of thrombi in acute ischemic stroke is associated with HARM. Additionally, leukocyte subtype composition, together with fibrin and red blood cell content, may potentially contribute to the underlying pathophysiology of stroke. Our findings suggest a potential association between early BBB disruption, as indicated by HARM, and the predominant structural components of the thrombus, which may have implications for post-stroke neuroinflammatory responses.

These observations should be considered hypothesis-generating and require validation in larger, adequately powered studies with prespecified analytical frameworks. Future investigations integrating detailed thrombus analysis with advanced neuroimaging may help clarify the pathophysiological mechanisms underlying acute ischemic stroke.

## Data Availability

The original contributions presented in the study are included in the article/Supplementary material, further inquiries can be directed to the corresponding author.
