# Supplementary material for: Hyperintense acute reperfusion marker (HARM) and thrombus analysis in acute ischemic stroke
Source: Front Neurol. 2026 Mar 19;17:1723099. doi: 10.3389/fneur.2026.1723099 (PMC13045561; doi:10.3389/fneur.2026.1723099)
Supplement: Supplementary file 1 [file Data_Sheet_1.pdf]

SUPPLEMENTARY MATERIAL

Hematoxylin & Eosin Staining

All thrombus samples were fixed with 10% formaldehyde solution. After fixation, tissue processing was done with formaldehyde-ethylene-xylene-paraffin by using automated tissue processing device (Tissue-Tek VIP 6 Al, Sakura Finetek USA). 4µm sections were prepared from paraffin blocks and taken onto slides. H&E staining was performed following the protocol as deparaffinization (incubation at 60 °C for 20 minutes and then xylene), alcohol, hematoxylin, washing with water, acid alcohol, lithium carbonate, alcohol, eosin, alcohol with increasing concentrations. H&E-stained slides were reviewed and images were obtained by for analysis.

Immunohistochemical Staining

In immunohistochemical stainings, CD3, CD20 and CD45 stainings were performed to determine T and B cell and total leukocyte counts, respectively. Automated staining system (Dako Omnis) was used for immunohistochemical staining. 3µm thick sections were taken from paraffin embedded thrombus samples.

For deparaffinization, thrombus sections were incubated with Clearify Cleaning Agent at 25 °C and then washed with distilled water for 5 seconds. The sections were then incubated for 30 minutes at 97°C with EnVision FLEX Target Retrieval Solution (50x, pH:9, Dako Omnis) for CD20 and CD45 stainings and EnV FLEX TRS (50x, pH 6.1, Dako Omnis) for CD3 staining for antigen retrieval. All sections were incubated with CD3 (GA503, Dako Omnis), CD20 (GA604, Dako Omnis), CD45 (GA751, Dako Omnis) antibodies for 20 minutes, and then washed with wash buffer for 2 minutes. After blocking with EnVision FLEX Peroxidase-Blocking Reagent for 3 minutes and incubation with Rabbit Linker for CD3 staining, Mouse Linker for CD20 and CD45 stainings for 10 minutes and washed with wash buffer for 2 minutes after each step. Sections were incubated with EnVision FLEX/HRP polymer for 20 minutes and washed 3 times for 2 minutes with wash buffer and 30 seconds with distilled water. All sections were incubated with EnVision FLEX DAB for 20 minutes and then washed for 5 minutes with Dab substrate buffer and 2 minutes with wash buffer. Afterwards, the sections were incubated with Hematoxylin (Dako Omnis) for 4 minutes and then washed with distilled

water for 2 minutes and washing buffer for 2 minutes and counterstained. Images were obtained after the sections were examined under the microscope and the digital slides were scanned.
